# The Dangers of Chloroform

**Published:** 1877-07

**Authors:** 


### ARTICLE IV.

### *The Dangers of Chloroform.*

The trial of the Rahway dentist for causing the death of Walter E. Lewis with chloroform has attracted a great deal of attention, not only on the part of the public, but of the profession. As will be remembered, last January the boy, shortly after having eaten a hearty supper, called upon the dentist for the purpose of having a tooth extracted. Chloroform (slightly mixed with ether) was administered while the patient was sitting in a chair. After a few inhalations with the napkin close to the face, the pulse began to flag, when the tooth was hurriedly extracted. The child's head then rolled to one side, and respiration ceased. The patient was left in the chair for several moments without any attempt at resuscitation, and until a neighboring physician arrived. Attempts at artificial respiration were then made, the galvanic battery applied, and all the other means usually employed on such occasions, but to no purpose.

The post-mortem examination revealed all the organs in a healthy condition. The heart was free from fatty degeneration, and was flabby; the right side contained a small quantity (half an ounce) of dark fluid blood, while the left side was entirely empty. The blood was dark and fluid. As far as could be judged from the autopsical lesions, the victim was in good health at the time of the accident. The coroner's jury brought in a verdict against the dentist, charging him with gross carelessness in the administration of the anæsthetic. The Grand Jury sustained the charge and the case was regularly tried in the court of Oyer and Terminer. The charge was manslaughter.

The prosecution endeavored to prove that the defendant did not use the ordinary precautions in giving the anæsthetic; that he was intoxicated at the time; that not a sufficient amount of air was allowed, and that the death was directly caused by such acts of carelessness. It was shown that the child had a full stomach at the time of taking the anæsthetic, that neither the pulse nor the respiration was watched during the administration, that the tooth was drawn when the patient was in a dangerous condition, and that the patient was left in the chair after the accident for several minutes without help.

As might have been anticipated, the charge of drunkenness could not be satisfactorily sustained. The experts for the prosecution stated that it was improper to administer chloroform while the patient had a full stomach, or was in a sitting posture; that it was always the rule to examine the chest organs; and, in case of doubt, the stomach; that a full stomach and the upright position invited accident; that a failure of the pulse and respiration demanded an immediate resort to artificial respiration.

The main argument of the defence was based upon the fact that death not infrequently occurs during the different stages of anæsthesia without any apparent cause, without any better explanation than that afforded by the vague term, idiosyncrasy. It was also proved that the defendant was quite accustomed to give chloroform, and that he administered it in the usual manner for dental operations.

The judge in delivering his charge stated that, the burden of proof resting upon the State, the benefit of a doubt belonged to the defendant. Reciting the well-known aphorism that a practitioner is only expected to exercise ordinary skill in the practice of his profession, the question was one of degree rather than kind, and was, under the circumstances, to be settled by the jury. The ruling was to the effect that a practitioner was not accountable for the death of a patient which death might have been prevented by extraordinary skill on the part of another. This was the charitable construction which the law placed upon the want of skill possessed by the prisoner. Why this should apply in the case of so dangerous an article as chloroform does not appear, more especially when it was used contrary to the rules laid down by authority.

Although it could not be proved that death from chloroform was produced as the direct result of carelessness, the presumptive evidence of such a fact was very strong. A full stomach is not of itself a cause of death in chloroform, although few medical men would be willing to run the risk of its influence as an impediment to the action of the lungs or heart, especially when the patient is in an upright position. The impaction of vomited material in the trachea is another element of danger; but as no such accident occurred in this case, the consideration of its possibility, was for obvious reasons, not allowed. The law can really hold no one accountable for accidents which might have occurred from negligence. There must be a direct association between cause and effect. If the patient in question had died by suffocation as the result of food in the trachea, the full stomach would have been a more important factor in the case. The absence of any positive proof that the posture, full stomach, rapidity of administration, deficiency of atmospheric air, individually or collectively, had any positive and direct effect in producing death in this case, gave the defence a wide latitude for instilling doubt in the minds of the jury. The opinion of the medical experts was to the effect that the chloroform was carelessly administered, and that the fatal issue was thereby invited. By an oversight on the part of the prosecution, there was very little stress laid upon the neglect to revive the boy the moment the signs of danger appeared. Instead of attempting this the dentist ran for help, and when help arrived several minutes afterwards, the child was still upright in the chair. This at least does not prove the exercise of even ordinary skill in cases of danger. There was a reasonable chance that the child might have been saved if resuscitation had been resorted to at once. It is impossible to say how many lives might be sacrificed during anæsthesia, if every one followed the practice of the Rahway dentist.

The neglect to place the child in the recumbent position as soon as danger appeared, was the strongest point against the prisoner. There does not appear to be the slightest excuse for it. No one has a right to use an agent known to be treacherous and dangerous unless he is competent to exercise requisite skill in counteracting bad effects when they show themselves. If the dentist possessed such skill, he did not use it. Whatever may be said of the other circumstances of the case, the omission to perform so obvious a duty as placing the boy in a proper position immediately after the accident, and resorting to the ordinary means for overcoming asphyxia, syncope, etc., amounts professionally speaking, to criminal neglect. But, as we before remarked, very little notice was taken of this, and the defendant was acquitted.

The result of the trial does not help to establish any new principle in regard to the administration of chloroform in dental practice. Notwithstanding, twelve intelligent men in New Jersey have decided that no fault can be found with the administration in this case, the profession at large will still hold to its settled opinions, and still lean to the safe side, declining to follow so questionable an example.—*Editorial in Med. Record.*

